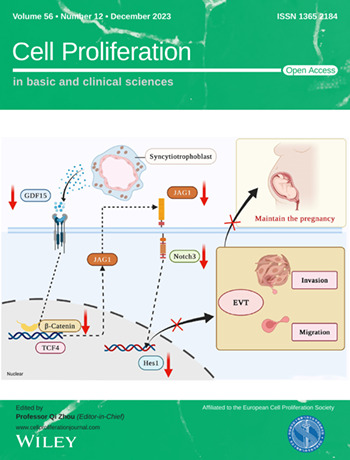# Additional Cover

**DOI:** 10.1111/cpr.13585

**Published:** 2023-12-02

**Authors:** Chunzi Lyu, Tianxiang Ni, Yaqiu Guo, Tingting Zhou, Zi‐Jiang Chen, Junhao Yan, Yan Li

## Abstract

The cover image is based on the Original Article *Insufficient GDF15 expression predisposes women to unexplained recurrent pregnancy loss by impairing extravillous trophoblast invasion* by Chunzi Lyu et al., https://doi.org/10.1111/cpr.13514.